# Editorial: Enaction and Ecological Psychology: Convergences and Complementarities

**DOI:** 10.3389/fpsyg.2020.617898

**Published:** 2020-11-26

**Authors:** Marek McGann, Ezequiel A. Di Paolo, Manuel Heras-Escribano, Anthony Chemero

**Affiliations:** ^1^Department of Psychology, Mary Immaculate College, Limerick, Ireland; ^2^Ikerbasque, Basque Foundation for Science, Bilbao, Spain; ^3^Center for Computational Neuroscience and Robotics, University of Sussex, Brighton, United Kingdom; ^4^IAS- Research, University of the Basque Country, San Sebastián, Spain; ^5^Department of Philosophy, Faculty of Philosophy, University of Granada, Granada, Spain; ^6^Filolab Unit of Excellence, University of Granada, Granada, Spain; ^7^Departments of Philosophy and Psychology, University of Cincinnati, Cincinnati, OH, United States

**Keywords:** ecological psychology, enaction, embodiment, agent-environment interaction, skill learning, mutualism

The past several decades in cognitive science have seen an increasing recognition of the importance of the body, and of the relationship between the body and the environment, to our understanding of the mind. Forms of this recognition have varied substantially, with some seeing it important to add a role for the body into existing computational and representational accounts of cognition (e.g., Clark, 2007; Shapiro, 2011; Barsalou, 2015), while others finding in the body a different approach altogether, one which produces quite a different picture of the mind than those accounts which have formed the mainstream and traditional forms in the cognitive sciences.

Some of these more radical forms of embodied cognitive science have developed fairly independently of one another, but nevertheless have come to share some core theoretical characteristics—accounts that emphasize the role of action for perception and that do not involve computation or representations in explanatory roles. In their place we find discussions of skilled bodily activity in providing accounts of the performance of cognitive tasks.

Two well-developed such approaches are those of ecological psychology, deriving substantially from the work of psychologist Gibson (1966, 1986), and that of enactive cognitive science, building largely on foundations laid by Varela et al. [1991; see also Thompson (2007)]. Both approaches have continued to expand and diversify in their accounts of psychological and cognitive phenomena, framing significant empirical and theoretical work within the cognitive sciences to date (e.g., Chemero, 2009; Rietveld and Kiverstein, 2014; Di Paolo et al., 2017, 2018; Hutto and Myin, 2017; Cummins, 2018; Heras-Escribano, 2019a; Turvey, 2019; Wagman and Blau, 2020).

These two approaches appear to share a number of key theoretical and methodological commitments, including a conception of cognitive activity as being performed in skilled engagement between an agent and a rich, complex world, and such accounts being couched in terms that do not depend on computational or other forms of representations for their explanatory power. Despite these shared commitments and other apparent resonances between the approaches, communication between these two groups of researchers has been surprisingly sparse, and collaboration more rare still. Though several authors (Chemero, 2009; McGann, 2014; Rietveld et al., 2018; Heras-Escribano, 2019b) have recommended some form of integration between them, just what such an integration would entail, and whether it might even be possible, has not been worked out in detail. Our primary aim in advancing this Frontiers Research Topic has been to provide a forum where such parallels, resonances, convergences, and complementarities, could be aired, and given proper consideration, in as fulsome a form as possible. That includes identifying tensions and incompatibilities and assessing whether they are merely superficial or harder to resolve. In a domain more richly illuminated by such a diversity of perspectives further work on development of either an integrated approach, or continued separate development, can be conducted with a richer understanding of the relationships between these two promising modes of cognitive scientific research.

The 30 papers that make up this Research Topic address a wide range of questions concerning ecological psychology, enactive cognitive science, and their shared domain of scientific interest. The topics broached bring to the fore a number of key points of contact between ecological and enactive thinking, and provide varying evaluations for the possibility of some kind of reconciliation, complementarity, or alignment of the two. Some authors highlight divergence, conflict, or even distinct foundations, which motivate a pessimistic prognosis on integration, noting differing views on the relationship between the agent and the world, or sometimes even the basic scientific approach. Others appear more optimistic that these are perhaps two perspectives on the same avenue of scientific advancement. Even in this latter case, however, it is clear that the differences between the two are not simply ones of appearance, but potential points of theoretical dissonance that will require real theoretical or empirical work if they are to be reconciled. In this collection of papers we see a number of potential diagnoses of differences, ranging from different starting points in examination of the agent-world relationship, to different commitments to “realism” about the world, or the role of other agents in our account of human cognition, where specific gears of ecological and enactive theories touch one another and either grind hopelessly or engage with some degree of success.

Though there are a number of themes or threads we might recognize as connecting the set of papers in this Research Topic, in what follows we outline a scheme with a few broad strands. One recurring theme is the foundational question of the relationship between the agent and the world; how it might best be understood, represented, and the varying implications that follow. A second theme is that of skill learning and the dynamics of attunement between agent and world. Regardless of how the relationship between agent and world is conceived, it is recognized as dynamic and vital. How this dynamism is to be considered and understood affects how we approach questions of cognitive science, highlighting some particularities that either strengthen or undermine the apparent consonance of ecological and enactive approaches. A third theme, unpacking that relationship in more specific terms, is the complex topic of affordances. Introduced by Gibson (1977, 1986), it has since its beginning proven to be as contested as it is useful. The frequency with which it is deployed bespeaks its value both in formulating and executing empirical research agendas as well as articulating and problematising the different ways in which different approaches formalize the agent-environment relationship. Finally, while all of these themes involve a recognition of the central role played by the active body, some contributions elaborate in more detail aspects of embodiment that are relevant to elucidating the relations between the two approaches, such as the roles of agency, of embodied experience, and of the brain, as well as different forms of embodiment.

A key tenet that both approaches appear to share is that of a reciprocal relationship between agents and their environment. Cognition arises within that relationship, rather than, say, entirely within the head of the agent in question. The multiple and entwined circularities of this relationship are highlighted by Fuchs, who examines mutual causal and dynamical relations in the structures of situated embodiment. The dynamic, active environment that these circular relations imply is on the face of it a point of clear agreement between ecological and enactive approaches, which separates them distinctly from mainstream perspectives. A number of authors identify dissonances in the particulars of how this relationship should be analyzed and understood, and there is a clear diversity of positions taken on this ostensibly common ground, even when it comes to how such a relationship should be discussed by us scientists in our practice.

Given the foundational role of the agent-environment relationship in both approaches, it seems vital to make any differences in its conception explicit and to examine their implications. Heft notes some apparent incompatibilities based on seemingly different roles for sensation and action in the agent-environment relationship. A somewhat similar diagnosis, though with a more optimistic prognosis, is offered by Read and Szokolszky. Providing a valuable historical perspective, Feiten explores how researchers from the two approaches seem to draw from different descriptions of von Uexküll's notion of the *Umwelt* to make sense of mutualism or reciprocality. These examinations offer key insights into how questions are framed differently by different researchers. What is more, such foundational concerns extend beyond the traditional boundaries of the cognitive sciences. If the relationship between agent and environment is as complex as ecological and enactive approaches imply, then the ramifications affect not just cognitive theory, but scientific practice more generally, explored by Cummins, as well as our conception of the person, our ethical obligations, and participation in society, an issue broached by de Pinedo García. Resources for consideration of the complementarity inherent in this mutualism between agent and environment may indeed send us further afield from mainstream cognitive science; McKinney notes the possible value of the work of various Japanese philosophers in engaging with the topic, and finding a path toward continuing fruitful interactions between the two approaches without prioritizing or undermining either.

Unpacking and systematically elucidating this concept of mutualism clearly provides plenty of work to do. Nonaka explores the inexhaustible richness of the environment, finding there a texture sufficient to account for all aspects of the contact between agent and world, in doing so, seeing off any concerns regarding “constructivism” sometimes perceived in enactive approaches. Crippen also examines this perceived incompatibility of ecological psychology's “realism” vs. enactivism's “constructivism.” Examination of the relationship between agent and world suggests that this dichotomy is not as threatening as it might seem. McGann similarly addresses this friction between both perspectives, and in an insight shared with several papers in the collection, notes how the complexity of interacting processes over different timescales dissolves some of the apparent disagreement, though leaving work still to do.

While it is important to notice broad theoretical divergences, and work toward clarifying their significance and hopefully resolving them, several authors take on a more concrete stance and attempt to work out differences and complementarities between the two approaches in the case of more practical issues. One such recurring topic concerns the development and learning of action and perception skills. These involve what Di Paolo calls *transactional* couplings in his overview of pictorial representations of the relation between agent and environment, and which he identifies as being a research area of significant historical overlap between the two schools. Baggs et al. notice some tensions between the enactive and ecological conceptions of skill learning. Moving beyond perspectives that pin skills to the body, they proposed an extended unit of analysis in the organism's situated activity and the self-organization and constraints that emerge in this activity. The move is analogous to the proposal presented by Corris, who offers a developmental answer to the question of the specification of the environment, which she finds unsatisfactorily treated by both the enactive and ecological perspectives: why do certain contingencies matter and not others? The idea of a developmental niche successfully combines ecological and enactive sensitivities and serves as an example of the kinds of theoretical advances we wish to see. A complementary notion to that of the developmental niche is perhaps James's notion of *enhabiting*, the process of individuation by which the shared complex of a species-typical habitat (from the point of view of us scientists) is enacted as an Umwelt for an individual organism. Building on the work of Simondon, James describes a process akin to equilibration (Di Paolo et al., 2017) by which a specific agent-environment system brings activities at multiple timescales into coherence with one another. A similar dynamic of reconciliation or coordination is outlined by Sepúlveda-Pedro in his contribution, this time in terms of norms. He raises the question of normativity contrasting the enactive approach with the skilled intentionality framework (Rietveld et al., 2018) and their respective views that norms are enacted by agents and that agents attune to pre-existing norms. These views, again, can be reconciled by adopting a developmental perspective that appeals to the work of Merleau-Ponty. Drawing observations from cases in sports psychology, Avilés et al. also see the complementarities between enaction's attention to bodily experience and ecological clarifications of skill acquisition as calibration and the education of attention and intention. de Carvalho and Rolla also address the question of learning, this time in terms of the highly contested idea of information. Offering a distinctly optimistic view on the compatibility of different conceptions of the idea extant in the cognitive scientific literature, they provide examples of how direct learning may be understood as sensitivity to information about the likely outcome of particular actions.

Putting the focus on the microgenesis of specific skills reveals even richer links between the approaches. In their detailed analysis of dynamic touch, Travieso et al. find clear complementarities between ideas of sensorimotor contingencies and information detection through active exploration. In this way, they touch again on the question of an agent's activity, which is also empirically explored in a sensory substitution study of haptic perception by Froese and Ortiz-Garin. Using the Enactive Torch in a double participant set-up with active and passive conditions, the authors find that the role of agency in perception appears to be only instrumental. This is in line with how self-generated activity has been conceived historically in ecological psychology. Bermejo et al. discuss this history by examining the changing reception of the work of neuroscientist Richard M. Held, who pioneered studies that revealed the importance of voluntary activity in perceptual learning. While James Gibson seemed to think voluntary activity was merely facilitatory of processes that could occur otherwise, Eleanor Gibson and colleagues working on perceptual development thought it played stronger enabling roles. Enactivists would agree and argue that they can even play constitutive roles. The authors discuss the difficulties of taking the active/passive distinction as binary, and offer a series of practical dimensions for characterizing self-generated activity.

The notion of affordance is perhaps the best known of Gibson's contributions to theory, and also the most contested. The concept, having been introduced as something that is “neither an objective property, nor a subjective property; or it is both, if you like” (Gibson, 1986, p. 129), seems to offer a means of articulating and perhaps formalizing the coupling between agent and environment. What is more, it does so in a way that motivates empirical work. In several papers here, affordances are deployed as a lens to bring certain points of contact between the ecological and enactive approaches into focus and examine them. Affordances prove useful to others more in terms of their potential to speak coherently about recurring themes in the dynamics between agent and environment that occur at different levels of analysis, or in apparently different domains. Cognitive science sprawls across the entire realm of human and non-human life, with researchers on one hand examining the relationship between moving objects and bodily motion, and moving artworks and therapeutic empathy on the other, to mention just two of innumerable possible landmarks in this rich landscape. Affordances, broadly construed, offer a means of approaching these apparently disparate domains in a coherent manner. While some are concerned this threatens to dilute the notion to the point of vacuity, others use the concept to make sense of some of the richer and more complex aspects of human existence in a way that illustrates important continuities between what are traditionally seen as distinct fields. What is more, these insights help to articulate points of tension or resonance between ecological and enactive approaches, and set out some of the work that needs to be done if the two are to come to occupy a common scientific ground.

Gastelum addresses some of the complexity regarding this range of domains from the point of view of temporality: that affordances must occur at a range of temporal scales, and be accounted for accordingly. Attentive to issues of scale, Loaiza et al. similarly examine the complexity of the domain of human activity, and use affordances to approach the interplay between temporal scales that helps make sense of their continuity. This more liberal notion of affordances would seem to offer something akin to a theoretical invariance, a means of thinking of the agent-environment system in coherent terms whether the discussion refers to a cell in a chemical gradient or a person in a conversation. Following a multiscalar perspective, Trasmundi and Cowley present an ethnographic study of the processes of reading and imagining. Their enactive-ecological approach encompasses saccadic eye movements, interaction with cognitive artifacts (such as books), vocalizations, and multi-modal social interactions, demonstrating again the purchase of examining complex cognitive phenomena over a range of scales. Brancazio warns that such continuities should not be oversold, however. Also using affordances as the theoretical tool, she attempts to lever apart the domains of physical and social activity, highlighting ethical implications that must be recognized and addressed where affordances are interpersonal, distinguishing them from a more basic reading of agent-environment relation. Caravà and Scorolli's intervention suggests that the concept of affordances may be effectively deployed in the empirical study of affective or emotional aspects of life, examining the ways in which projectible properties of the visual world are encountered in terms of their social and cultural value. The role of social, cultural factors in organizing and giving valence to affordances is addressed by Harrison in his micro-ethnographic study in a commercial setting. He seeks to embed the enactive conception of sense-making within a framework informed by the ecological psychology of Barker, Schoggen, and others (see Barker, 1968; Schoggen, 1989), literally “exploring” the various forms of affordances created as part of a marketing campaign behavior setting within a shopping center in Hong Kong.

Questions about the body run through most of the contributions: the body as an active agent, the body as situated, the body in regards to others, and the embodied character of perception and experience. These are general zones of convergence between the approaches. Some contributions elaborate on these ideas. Segundo-Ortin contrasts the enactive and ecological approaches to embodied agency and argues for the benefits of adopting a dual approach that combines enactive accounts of sensorimotor equilibration with an ecological focus on how perceptual information contributes to the actualization of sensorimotor habits. The analysis of embodied agency also preoccupies Popova and Raczaszek-Leonardi, who discuss dissimilarities and complementarities between the two camps by drawing on the phenomenology of lived bodily experience. The practical implications of foregrounding agency and lived experience are well-exemplified in the ecological-enactive model of disability presented by Toro et al.. The authors demonstrate that concepts of disability are not exhausted by physiological or medical normativity, but demand the constitutive role of lived experience. Through qualitative interviews with patients with cerebral palsy, they show that their experience can demonstrate tendencies toward maximal grip, and therefore need not, in all cases, be considered as arising from a “pathological embodiment.”

Insistence on the importance of embodiment has been, and continues to be, a point of contrast with neurocentric perspectives still prevalent in cognitive science. In turn, the question may be put to both enactivists and ecological psychologists: What about the role of the brain in these theories? No one denies that the brain plays crucial roles in explaining cognition, and enactive researchers have offered explicit non-representational theories about what this role could be (e.g., Varela et al., 2001; Fuchs, 2018). Other theories, such as coordination dynamics and neural reuse, can also meet both enactivist and ecological theoretical constraints (e.g., Kelso et al., 2013; Anderson, 2014). Ryan and Gallagher discuss and compare some of these ecological-enactive proposals, in particular, apparently convergent conceptions of the brain as a resonant, rather than representational organ, and they examine whether these conceptions are metaphorical or can offer specific mechanisms. Cashing in on the resonance of these and other enactive-ecological ideas (such as that of attunement) with musical performance, they suggest that activities such jazz improvisation provide rich case studies for combined enactive and ecological theories of brain function (and environmentally situated bodily activity).

It is clear that this and the many other questions examined in this Research Topic are ripe for further research. As with jazz performance, we are happy to observe that the contributions do not follow a single orchestrated pattern. Voices rise and recede, sometimes performing duets, sometimes trios, with attention to history but without entrenching in it, also with interesting innovations and an element of unpredictability signifying at least that the road ahead remains open. What is important, in our view, is that the conversations have started and we are certain they will continue.

## Author Contributions

MM and ED drafted the Editorial and the rest of the authors contributed to its completion. MH-E and ED organized the workshop that triggered the preparation of this Research Topic. All the authors have worked substantially on the conception of this Research Topic and during the editing process.

## Conflict of Interest

The authors declare that the research was conducted in the absence of any commercial or financial relationships that could be construed as a potential conflict of interest.
